# Correlations between psychological anxiety symptoms and physical anxiety symptoms in dental anxiety—a cross-sectional study with 1327 patients

**DOI:** 10.3389/froh.2025.1612982

**Published:** 2025-08-28

**Authors:** Laura Agnes Ingrid Magerfleisch, Nertsa Cunoti, Rezart Qorri, Katharina Marilena Weil, Hannah Tröger, Juliane Häring, Lisa Irmscher, Katja Petrowski, Hendrik Berth

**Affiliations:** ^1^ Research Group Medical Psychology and Medical Sociology, Division of Psychological and Social Medicine and Developmental Neurosciences, Medical Faculty Carl Gustav Carus, Technische Universität Dresden, Dresden, Germany; ^2^ Department of Medical Psychology and Medical Sociology, University Medical Center of the Johannes Gutenberg-University Mainz, Mainz, Germany

**Keywords:** dental anxiety, dental anxiety scale, dental fear survey, oral health, anxiety symptoms, cross-sectional study, Germany

## Abstract

**Background:**

Dental anxiety is a prevalent phenomenon with the potential to impact both psychological well-being and oral health outcomes. It may lead to individuals avoiding treatment. This study aims to explore the relationship between psychological anxiety symptoms and physical anxiety symptoms in dental anxiety.

**Methods:**

Between 2019 and 2022, a cross-sectional study was conducted including 1,327 patients from a variety of dental practices in Germany and students (age range: 18–85 years; mean: 39.65 years, 60.7% female). Participants completed validated questionnaires, including the Dental Anxiety Scale (DAS), the Scale Somato-visceral arousal of the Dental Fear Survey (DFS-SVA), the Brief Symptom Inventory 18 (BSI-18 GSI), and the Oral Health Impact Profile (OHIP-5). Data was analysed using Mann–Whitney-U-tests, Spearman correlations, Kruskal–Wallis-tests and linear regression models. The significance level was set at *p* = 0.05.

**Results:**

The mean value for dental anxiety (DAS) was 9.81 (SD = 4.07) and for physical anxiety symptoms (DFS-SVA) 10.51 (SD = 4.70). Of the participants, 808 (60.9%) reported no dental anxiety, 368 (27.7%) were somewhat anxious and 151 (11.4%) reported severe dental anxiety. A strong correlation was observed between DAS and DFS (*r* = 0.544), while moderate correlations were found between DAS and BSI-18 (*r* = 0.300) and between DAS and OHIP-5 (*r* = 0.371). The application of regression analysis indicated that DFS-SVA was the strongest predictor of DAS scores (*β* = 0.434, *p* < 0.001), followed by BSI-18 GSI (*β* = 0.285, *p* < 0.001) and age (*β* = 0.174, *p* < 0.001). Gender was not found to have a significant influence on results. A significant disparity was observed in the results between male and female scores and on the DAS, DFS-SVA, and BSI-18 GSI compared to men.

**Conclusion:**

The study corroborates substantial correlation between psychological dental anxiety and physical anxiety symptoms. These findings underscore the necessity for early detection and targeted interventions within dental practices with the aim of enhancing both oral health and overall quality of life. It is recommended that future research endeavours focus on the investigation of causal relationships and the identification of effective treatment strategies to further enhance patient care.

## 1 Introduction

Dental anxiety is a prevalent phenomenon characterized by an apprehension of dental treatment. On a global scale, 15.3% of adults experience any form of dental anxiety, while 12.4% of adults demonstrate high dental fear or anxiety. Severe dental anxiety has been observed in 3.3% of adults (1). Dental anxiety is a recognised mental illness that can range from mild discomfort to extreme phobia. It has both psychological and physical implications for the affected (2). The condition is associated with a variety of symptoms. Consequently, patients exhibit a range of physical, cognitive, emotional and behavioural responses (3). The symptoms experienced by these individuals include sleep disorders, constant nervousness and concentration problems (4). Avoidance is the predominant method of coping with dental anxiety, leading to a vicious cycle (5). Deterioration in oral health has been shown to be a contributing factor to an increase in dental anxiety (6). The relationship between dental anxiety and treatment avoidance has far-reaching consequences. Several studies have demonstrated that anxious or phobic patients are more prone to experiencing untreated caries (7–9) and tooth loss (7, 9). In addition to the consequences regarding oral health, these patients may also exhibit a reduced general sense of well-being and a deteriorated quality of life (2). Dentists can mitigate levels of dental anxiety through the implementation of behavioural interventions (10, 11). It is therefore vital to recognise dental anxiety at the earliest opportunity. The manifestation of symptoms associated with physical dental anxiety may facilitate this process.

There are established instruments for measuring dental anxiety, such as the Dental Anxiety Scale (DAS) (12–14) and the Dental Fear Survey (DFS) (12). The Somato-visceral arousal of the DFS is an instrument that provides additional information on avoidance behaviour and physical reactions (15).

Despite extensive research on the prevalence and general characteristics of dental anxiety, the way anxiety symptoms manifest and interact remains insufficiently understood. In particular, the relationship between psychological components and physical symptoms has received limited empirical attention. While existing studies have identified significant associations between somatisation and dental fear (16), as well as correlations between psychological distress and dental fear (17), few have explored how these symptoms interact. This represents a research gap as a better understanding of the interplay between psychological and physical anxiety symptoms in dental anxiety incorporating the utilisation of instruments such as the DAS and the DFS, could enhance both diagnostic precision and the development of tailored intervention strategies.

Therefore, we aim to ascertain the correlation between psychological and physical anxiety symptoms in dental anxiety in adults. We assume a significant positive correlation. Furthermore, we hypothesise that higher levels of dental anxiety and its associated symptoms are linked to increasing general psychological stress, as measured by BSI-18.

## 2 Methods

The correlation between psychological anxiety symptoms and physical anxiety symptoms in dental anxiety has been evaluated using a retrospective study. A dataset of earlier studies involving a total of 1,327 adult patients was investigated.

The patients were requested to complete the questionnaires in the waiting room prior to undergoing treatment, following the provision of written informed consent. All respondents, who were required to be at least 18 years of age, were informed in writing about the objectives of the study. Participants were required to demonstrate a good command of the German language and an oriented appearance at the time of the survey, which was assessed by clinic staff. The questionnaires comprised the Dental Anxiety Scale (DAS) and the Scale Somato-visceral arousal of the Dental Fear Survey (DFS-SVA). Perceived oral health was recorded using the Oral Health Impact Profile (OHIP-5). The mental health of the participants was assessed by means of the Brief Symptom Inventory questionnaire (BSI-18).

The study was conducted in full accordance and approved by the Ethical Commission of the Technische Universität Dresden (protocol no. 232062011/29.07.2011).

### 2.1 Dental anxiety scale (DAS)

The Dental Anxiety Scale (DAS) is an instrument for the assessment of dental anxiety (12, 18). The DAS comprises four questions, which each question having five possible answers (13). The second to fourth question, for example, “When you are waiting in the dentist's office for your turn in the chair, how do you feel?”, relates to the respondent's feelings during various dentist-related situations (19). As the response options are ranked in ascending order from 1 to 5, the severity of dental anxiety can then be categorised within the range of 4–20 (20). The higher the score, the higher the dental anxiety. The patients were then categorised into three groups: little to no anxiety (score ≤10), somewhat anxious (score <10 ≤ 15) and severe dental anxiety (score >15). The reliability of DAS in other German studies was r_tt_ = 0.94 (12), whereas in this survey it is good (McDonald's Omega = 0.925).

### 2.2 Dental fear survey (DFS)

The Dental Fear Survey (DFS) depicts behavioural and physiological responses to dental anxiety (19). It was also used in German translation, analogous to the original by Kleinknecht et al. from 1973 (15). The content of the 20 questions is divided into three areas: Avoidance of dental treatment, physical reactions to the dental treatment and fear aroused by different dental procedures (15). The DFS responses are measured on a scale from 1 (none) to 5 (great) (21) for questions such as „When having dental work done, I feel nauseated and sick to my stomach” (19). In summary, the scale ranges from 20 to 100 (22). Scores under 51 indicate no dental fear, values between 51 and 75 reflect low anxiety, and scores over 75 display high dental fear (12). In this study only the scale Somato-visceral arousal during dental treatment (physical reactions) of the DFS (DFS-SVA) was used. The scale is comprised of five items including muscle tension, perspiration and nausea (19). The reliability of DFS in German studies was found to be r_tt_ = 0.97 (12) and in this survey the reliability of DFS-SVA is good (McDonald's Omega = 0.9).

### 2.3 Brief symptom inventory 18 (BSI-18)

The Brief Symptom Inventory 18 (BSI-18) is a tool for measuring psychological stress in patients with various mental and somatic illnesses (23, 24). The questionnaire is divided into three scales of six questions (somatization, anxiety, depression), and is then summarised to the total BSI-18 score (GSI) (25). In this study, only the GSI reflecting the general psychological stress was used (BSI-18 GSI). The items, for example, “feeling lonely” (23), are answered based on the patients’ experiences over the preceding seven days, offering five possible answers ranging from “not at all” (0) to “extremely” (4) (26). The total BSI-18 score ranges from 0 to 72, while each group can score between 0 and 24 (23). The reliability of the GSI of BSI-18 in other German studies was Cronbach's *α* = 0.93 (23), of BSI-18 GSI in this survey it is good (McDonald's Omega = 0.818).

### 2.4 Oral health impact profile (OHIP-5)

The Oral Health Impact Profile (OHIP-5) was developed in 1994 by Spade and Spencer (27). An analogous German version was used (28). It is a tool designed to measure oral health-related quality of life, and inquiries into four dimensions: oral function, orofacial pain, orofacial appearance, and psychosocial impact (29). The scale comprises five questions, with one item representing each dimension and an additional item for oral function (30). The questions, for example “Have you experienced difficulty chewing food due to oral problems?”, are answered on a five-point scale ranging from “never” (0) to “very often” (4) (31). The total score ranges from 0 to 20 with higher scores indicating a greater negative impact of oral health issues on an individual's quality of life (32). The reliability of the OHIP-5 in other German studies was found to be Cronbach's alpha = 0.79 (28), in this survey it is good (McDonald's Omega = 0.904).

### 2.5 Statistical analysis

The data was analysed using the program IBM SPSS 30. The Kolmogorov–Smirnov test was used to determine whether the data was normally distributed. To facilitate the comparison of groups, Mann–Whitney-*U*-Tests and Kruskal–Wallis-Tests were calculated. The magnitude of the effects was reported using Rosenthal's r or Eta^2^ respectively. Furthermore, Spearman correlations and a multiple linear regression analysis were conducted. The statistical relevance was determined using the *p*-value at *p* = 0.05. The reliability of the scales used was determined using McDonald's omega.

The required sample size for a robust statistical analysis was calculated using the G*Power 3 software program (33). For the implementation of a Mann–Whitney–*U* test (a comparison of two groups) with a predefined effect size of d = 0.05, a significance level of *p* = 0.05 and a power of 95% (1−*β* = 0.95), a minimum of 92 participants per group (total *n* = 184) are required. For one-way analysis of variance (ANOVA, comparison of three groups), with a predefined effect size of f = 0.25, a significance level of *p* = 0.05 and a power of 95% (1−*β* = 0.95) *N* = 252 individuals are required, as there is no corresponding function for the Kruskal–Wallis-Test. The required sample size for Pearson correlations is *N* = 115 individuals [predefined effect size of *p* = 0.30, significance level of *p* = 0.05 and a power of 95% (1−*β* = 0.95)] since there is no corresponding function for Spearman correlations. For multiple linear regression analysis with four predictors, the required sample size is *N* = 129 [predefined effect size of f = 0.15, significance level of *p* = 0.05, power of 95% (1–*β* = 0.95)].

## 3 Results

In the period between 2019 and 2022, a survey was conducted in various dental practices in Germany, encompassing a total of 1,327 students and patients (see overview in Table 1). The age of the respondents varied between 18 and 85 years, with a mean of 39.65 years (SD = 15.94). The proportion of male participants was 38.9% (514), while the proportion of female participants was 60.7% (803). The remaining 0.5% (6) had a different gender. Most respondents stated to have a high school diploma, such as Abitur or acc. university entrance qualification (57.6%, 586). A further 31.7% (323) of participants had an intermediate school-leaving certificate. Of the remaining patients, 8.2% (83) had a lower secondary school-leaving certificate, 1.6% (16) had another school-leaving certificate and 1.0% (10) had no school-leaving certificate.

**Table 1:** Overview of the studies includes in the analyses.

Study no.	Survey period	*N*=	Sex: female *N*, %	Age Range, M (SD)	Level of education: high school diploma or higher *N*, %	Description
1	December 2019–July 2020	132	89 (67.2)	18–80 42.28 (15.31)	39 (29.8)	Survey among patients at a dental practice (26)
2	January–June 2020	102	60 (58.8)	18–77 40.30 (15.74)	23 (22.5)	Survey of patients in two oral surgery practices
3	November 2019–March 2020	307	172 (56.0)	46–54 47.15 (0.53)	116 (37.8)	Population based sample, Wave 31 of the Saxon Longitudinal study (47)
4	October 2021–March 2022	398	278 (70.6)	18–80 28.02 (11.19)	329 (82.9)	Survey of students from Technische Universität Dresden and patients at various dental practices
5	July 2022–October 2022	92	33 (35.9)	19–67 25.64 (7.21)	92 (100.0)	Online survey among students at Technische Universität Dresden
6	September 2022–October 2022	296	171 (57.8)	18–85 50.54 (18.50)	103 (34.8)	Survey among patients at a dental practice (48)

N, sample size; M, mean; SD, standard deviation.

The mean value for dental anxiety (DAS), somatic anxiety symptoms (DFS-SVA), psychological distress (GSI BSI-18), and oral health-related quality of life (OHIP-5) are shown in Table 2. The Kolmogorov–Smirnov test demonstrated that the data is not normally distributed (DAS D = 0.130, *p* < 0.001; DFS-SVA D = 0.161, *p* < 0.001; GSI BSI-18 D = 0.169, *p* < 0.001 and OHIP-5 D = 0.185, *p* < 0.001). The descriptive analysis revealed gender-specific differences in nearly all the investigated variables (Table 2). A significant disparity in performance was observed between male and female subjects, with female subjects demonstrating higher DAS scores. A similar tendency was also observed in DFS-SVA. Furthermore, psychological distress, measured by the sum-score of BSI-18 (BSI-18 GSI), was found to be significantly higher in women than in men. The values in OHIP-5 did not differ significantly between women and men. The effect sizes are all small (*r* < 0.30).

**Table 2:** Descriptive values for DAS, DFS-SVA, BSI-18 GSI and OHIP-5, comparisons between men and women (Mann–Whitney-*U*-tests, *r* = effect size rosenthal's r).

Instrument	N	M	SD	Gender	N	M	SD	Mean difference	Std. error difference	95% CI lower	95% CI upper	*p*	U	z	r
DAS	1,317	9.81	4.07	Male	514	9.17	3.79	−1.05	0.23	−1.49	−0.60	**<0**.**001**	1,77,152.5	−4.354	0.120
				Female	803	10.22	4.19								
DFS-SVA	1,317	10.51	4.07	Male	514	9.87	4.16	−1.07	0.25	−1.57	−0.058	**0**.**002**	1,85,571.00	−3.101	0.086
				Female	803	10.94	4.99								
BSI-18 GSI	1,012	8.48	8.83	Male	376	6.70	7.05	−2.70	0.52	−3.73	−1.67	**<0**.**001**	97056.00	−4.661	0.147
				Female	626	9.41	9.41								
OHIP-5	1,317	3.25	3.61	Male	514	3.01	3.40	−0.38	0.19	−0.78	−0.00	0.090	1,95,118.50	−1.693	0.047
				Female	803	3.39	3.70								

Bold values indicate statistically significant results (*p* < 0.05).

N, sample size; M, mean; SD, standard deviation; p, *p*-value; U, U-value; z, z-score (standard score); r, correlation coefficient; DAS, dental anxiety scale; DFS-SVA, scale somato-visceral arousal of the dental fear survey; BSI-18 GSI, total Brief Symptom Inventory score; OHIP-5, oral health impact profile.

There were significant correlations found between all the variables studied (Table 3). The strongest correlation was observed between the somatic symptoms scale of the Dental Fear Survey (DFS-SVA) and the Dental Anxiety Scale (DAS) (*r* = 0.544). Further moderate correlations were identified between the BSI-18 GSI and the DAS (*r* = 0.300) as well as between the OHIP-5 and the DAS (*r* = 0.371). The lowest correlation was between the BSI-18 GSI and the DFS-SVA (*r* = 0.193).

**Table 3:** Spearman-Correlations between DAS, DFS-SVA, BSI-18 GSI and OHIP-5.

Instrument	Parameters	DFS-SVA	BSI-18 GSI	OHIP-5
DAS	*r*=	0.544**	0.300**	0.371**
	*p*	**<0**.**01**	**<0**.**01**	**<0**.**01**
DFS-SVA	*r*=		0.193**	0.277**
	*p*		**<0**.**01**	**<0**.**01**
BSI-18 GSI	*r*=			0.291**
	*p*			**<0**.**01**

Bold values indicate statistically significant results (*p* < 0.05).

DAS, dental anxiety scale; DFS-SVA, scale somato-visceral arousal of the dental fear survey; BSI-18 GSI, total brief symptom inventory score; r, correlation coefficient; p, *p*-value.

**Correlations that are statistically significant at the *p* < 0.01 level.

Multiple significant predictors for DAS scores were identified in Table 4. To detect the items influencing the extent of dental anxiety (DAS), a multiple regression analysis was conducted. The dependent variable (DV) was the DAS score. The independent variables (IVs) were age, gender, psychological distress (BSI-18 GSI), and somatic anxiety symptoms (DFS-SVA). The regression analysis demonstrated a significant predictive power (df = 4, F = 130.908, *p* < 0.001) and explained 34.0% of the variance (*R*^2^ = 0.343, adjusted *R*^2^ = 0.340). The strongest predictor was DFS-SVA, followed by BSI-18 GSI and age. The impact of the gender found to have no significant influence.

**Table 4:** Multiple regression analysis for dental anxiety (DAS), predictors: age, gender, BSI-18 GSI, DFS-SVA.

Predictor	Regression coefficient B	Std. Error	Beta	T	Sig.
(Constant)	2.984	0.470		6.343	**<0**.**001**
Age in years	0.041	0.006	0.174	6.729	**<0**.**001**
Gender	0.284	0.216	0.034	1.314	**0**.**189**
BSI-18 GSI	0.121	0.013	0.258	9.559	**<0**.**001**
DFS-SVA	0.372	0.023	0.434	16.371	**<0**.**001**

Bold indicates significant results.

Beta, standardised regression coefficient; T, t-value; Sig., significance (*p*-value); BSI-18 GSI, total brief symptom inventory score; DFS-SVA, scale somato-visceral arousal of the dental fear survey.

In the total sample, *N* = 808 (60.9%) participants were not anxious, *N* = 368 (27.7%) were somewhat anxious and *N* = 151 (11.4%) had a severe dental anxiety. The analysis of DAS in three groups revealed significant variations in all the parameters examined (Table 5). Individuals experiencing severe dental anxiety demonstrated significantly higher scores on the DFS-SVA, total BSI-18 GSI, and OHIP-5.

**Table 5:** Differences in DFS-SVA, BSI-18 GSI, OHIP-5 between groups with different 3 levels of dental anxiety (Kruskal–Wallis-tests).

Level of dental anxiety	N	M (SD)	Test statistic	*P*	Effect size
DFS-SVA					
Little to no anxiety	808	8.62 (3.43)	H = 355.972	**<0**.**001**	*η*² = 0.267
Somewhat anxious	368	12.27 (4.10)			
Severe dental anxiety	151	16.30 (5.54)	df = 2		
BSI-18 (GSI)					
Little to no anxiety	591	6.41 (6.62)	H = 93.816	**<0**.**001**	*η*² = 0.069
Somewhat anxious	299	9.55 (9.10)			
Severe dental anxiety	122	15.89 (12.47)	df = 2		
OHIP-5					
Little to no anxiety	808	2.23 (2.69)	H = 169.012	**<0**.**001**	*η*² = 0.126
Somewhat anxious	368	4.17 (3.73)			
Severe dental anxiety	151	6.40 (4.96)	df = 2		

Bold values indicate statistically significant results (*p* < 0.05).

N, sample size; M, mean; SD, standard deviation; H, test statistic; df, degrees of freedom; p, *p*-value; *η*², effect size; DFS-SVA, scale somato-visceral arousal of the dental fear survey; BSI-18 GSI, total brief symptom inventory score; OHIP-5, oral health impact profile.

DAS-Groups: ≤10: little to no anxiety, >10 ≤ 15: somewhat anxious, >15: severe dental anxiety.

## 4 Discussion

The aim of this study was to demonstrate the correlation between psychological and physical anxiety symptoms in dental anxiety. The large and diverse sample size of a general population utilised in this study (see Table 1) makes it particularly pertinent for dental practitioners.

The study demonstrated strong correlations between psychological dental anxiety symptoms (DAS) and physical dental anxiety symptoms (DFS-SVA). Consequently, a significant proportion of patients manifest a range of physical symptoms such as nausea and high heart rate, according to the severity of their dental anxiety. A smaller study in Germany in 2022 yielded comparable results (12).

The moderate correlation between BSI-18 and DAS indicates that patients experiencing elevated levels of general psychological stress also demonstrate a concomitant increase in of dental anxiety. Consequently, it can be hypothesised that mental stress could be a signal for possible dental anxiety. Several studies have demonstrated a strong correlation between dental anxiety and psychological distress, including depression, somatisation and generalised anxiety (17, 34–36). Accordingly, dentists should be vigilant for indications of dental anxiety in patients with documented psychological stress, with the objective of expeditiously identifying those experiencing dental anxiety.

The results also demonstrate a significant correlation between oral health-related quality of life (OHIP-5) and dental anxiety (DAS). Previous studies have shown that individuals experiencing high dental anxiety often perceive a greater negative impact of their oral health on their daily functioning and well-being and consequently have a lower oral health related quality of life (37, 38). As is thoroughly evidenced by the extant literature, the avoidance behaviour due to dental anxiety has a deleterious effect on oral health, reinforcing a vicious cycle in which worsening dental conditions exacerbate anxiety, which in turn results in further delays in treatment. This finding aligns with the conclusions drawn from earlier research (5). Nevertheless, these findings underscore the importance of early intervention and preventive dental care in managing dental anxiety.

Regarding gender differences, the present study found that women consistently reported higher levels of dental anxiety, physiological fear responses, and psychological distress than men. This was also shown in previous studies (17, 35, 39) and is consistent with existing literature suggesting that women are more likely to report anxiety-related disorders (40). The heightened anxiety experienced by woman can be attributed to a combination of emotional and social factors (41). However, the regression analysis revealed that gender was not a significant predictor of DAS scores. This finding suggests that while gender differences exist on a descriptive level, other factors such as psychological distress and avoidance behaviour play a more crucial role in determining the severity of dental anxiety.

This study suggests that dental anxiety increases with age. This contrasts with the results of other studies, which demonstrated a decrease in dental anxiety with increasing age (42–44) or identified middle age as the group with highest levels of dental anxiety (6, 45). The discrepancy may be attributable to sample characteristics. Two of the six surveys were conducted primarily among students, suggesting that the younger participants had a higher educational level compared to participants in the other groups. As dental anxiety has been shown to correlate with educational attainment (46), this difference in education level may have influenced the results.

The utilization of only validated instruments (see method section) is a crucial element in ensuring the reliability of this study. However, it is important to note that there are certain limitations must be considered. All instruments employed in this study were of a subjective nature and only stated by the patients. Despite the anonymity of the responses, the potential for societal pressures to influence the answers remains concern. Objective measures of dental health, such as the periodontal screening index (PSI) or DMF-T, could not be observed. Furthermore, over a period of several years, students and patients from different dental practices throughout Germany with all kinds of dental treatments were invited to participate in this study. Knowing that patients are more afraid of some interventions like extractions than of regular check-ups (41) may have had an influence on the answers to the questionnaires. Patients with high dental anxiety typically refrain from participating in oral-health interventions (5). Consequently, the representation of these patients in this study may be disproportionate. It is important to note that due to missing values for individual questions and/or individual survey waves, the study numbers are somewhat lower in some cases. Moreover, the cross-sectional nature of this study precludes the drawing of causal conclusions. It is recommended that future research should consider longitudinal studies with a view of enhancing our understanding of the causality and long-term development of psychological and physical dental anxiety symptoms.

Overall, the findings of this study demonstrate a close relationship between psychological dental anxiety and physical anxiety symptoms. It is imperative that dentists are cognisant of the psychological and physiological indicators of dental anxiety. In this way, anxious patients can be better recognized earlier, and treatments can be adapted better to individual needs.

## 5 Conclusion

The present study sought to confirm the correlation between psychological dental anxiety and physical anxiety symptoms. In addition, significant associations shown between dental anxiety (DAS), physical anxiety symptoms (DFS-SVA) and psychological distress (BSI-18 GSI), highlight a broader mental health impact of dental anxiety.

Further studies should be conducted to investigate the long-term development of dental anxiety and to establish detailed correlations between psychological and physical anxiety symptoms.

## Data Availability

The data presented in this study are available on request from the corresponding author. The data are not publicly available due to privacy issues.
